# Dietary acrylamide and AGE products in processed pet food: a review of the evidence and implications for safety thresholds in dogs and cats

**DOI:** 10.3389/fvets.2026.1970122

**Published:** 2026-10-22

**Authors:** Andreas Hopf, Matthew Muir, Alex England, Fariba Dehghani

**Affiliations:** 1 School of Chemical and Biomedical Engineering, University of Sydney, Darlington, NSW, Australia; 2 Pet Longevity Institute (PLI), Alexandria, NSW, Australia; 3 Lyka Pet Food, Sydney, NSW, Australia

**Keywords:** acrylamide, advanced glycation end products (AGEs), chronic disease burden, companion animal, dietary exposure and hazard

## Abstract

Traditionally processed pet foods, including kibble (dry), and retorted or canned formats (wet), are manufactured using high-temperature thermal processing methods that drive the formation of Maillard reaction products (MRPs) and acrylamide (AA). Dogs and cats commonly consume the same traditionally processed food as a daily dietary staple over prolonged periods, resulting in chronic, sustained dietary exposure to these compounds. In humans and rodents, dietary advanced glycation end products (AGEs) and AA are associated with diabetes, cardiovascular disease, neurodegeneration, and forms of cancer through mechanisms including oxidative stress, chronic inflammation, protein cross-linking, and activation of the receptor for advanced glycation end-products (RAGE). Dietary restriction of these compounds reduces disease markers in humans and rodents. Studies in companion animals indicate that dietary AGE intake influences systemic AGE exposure, and that mildly processed diets are linked to lower circulating AGE concentrations. Adverse health outcomes attributable to these compounds have not been demonstrated in companion animals. Unlike for human food, no regulatory benchmarks for acrylamide exist in pet foods, and no benchmarks for AGEs exist in any jurisdiction. This review examines the evidence on dietary AGEs and AA and health outcomes in humans and rodents, considers its potential relevance to companion animals, and discusses mildly processed diets as an alternative approach worth investigating.

## 1 Introduction

Traditionally processed pet foods, including kibble (dry) and canned or retorted (wet) formats, constitute the predominant diet for dogs and cats in developed countries (1). These products are manufactured using thermal processing methods such as extrusion, retorting, pelleting, and drying, which expose ingredients to temperatures ranging from approximately 90 °C to over 200 °C (2, 3). These processing techniques are employed to achieve microbiological stability, shelf-life, and palatability, but the high temperatures involved also drive the formation of Maillard reaction products (MRPs), including advanced glycation end-products (AGEs), and acrylamide (AA). Among these, AGEs and AA are increasingly associated with adverse health outcomes in humans and rodents (4, 5).

In humans, chronic dietary exposure to AGEs has been linked to a range of chronic diseases including diabetes, cardiovascular disease, neurodegeneration, and forms of cancer (5, 6). AA is classified as a probable human carcinogen (International Agency for Research on Cancer, IARC Group 2A) and is a recognized neurotoxin (7, 8). Evidence for comparable effects in companion animals remains limited.

As outlined above, dogs and cats commonly receive traditionally processed food as a consistent daily staple over prolonged periods, resulting in chronic, sustained exposure to MRPs and AA in contrast to the more varied human diet (1, 3). The potential health implications observed in humans and rodents, combined with the elevated and sustained dietary exposure documented in companion animals, raise an important question: should dietary intake of these compounds be addressed as a health concern in companion animals? Direct evidence in companion animals remains sparse, and causal links have not been established (Figure 1). This review examines the occurrence of dietary AGEs and AA in traditionally processed pet foods, summarizes the human and rodent evidence, and considers whether findings may be relevant to companion animal health.

**Figure 1:**
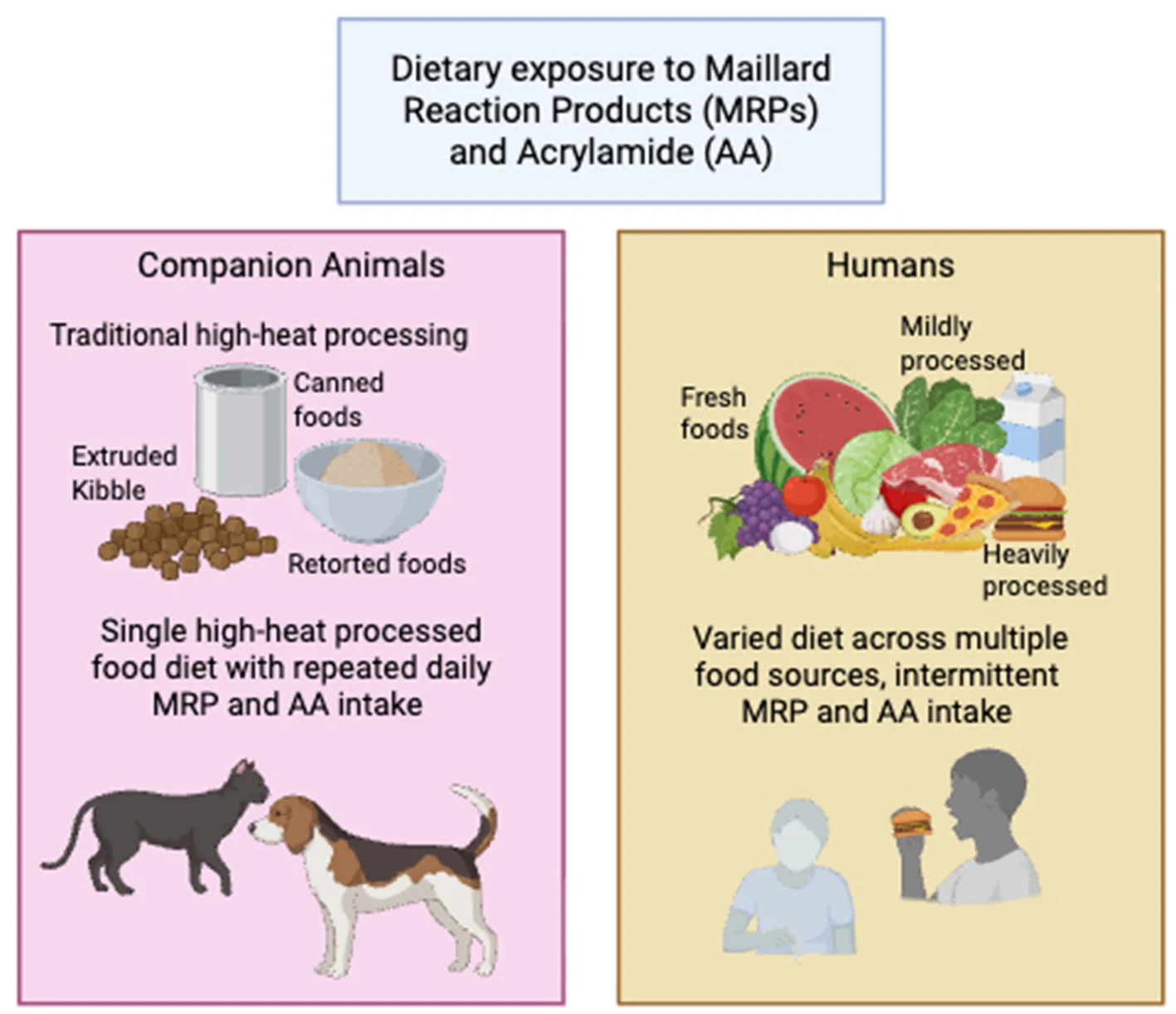
Dietary exposure to Maillard reaction products (MRPs) and acrylamide (AA) in companion animals and humans. Upper panels contrast exposure patterns, a repeated daily staple in dogs and cats against varied intermittent intake in humans. Lower panels follow proposed formation, absorption, mechanism and health outcomes, each stating the population in which the evidence was obtained. Solid arrows mark steps demonstrated in dogs and cats, dashed arrows steps inferred from human and rodent data. CML (Nε-carboxymethyllysine), CEL (Nε-carboxyethyllysine), HMF (5-hydroxymethyl-2-furfural), AGEs (advanced glycation end products) and RAGE (receptor for advanced glycation end-products). Created in BioRender.com.

## 2 Exposure to MRPS in pet food

### 2.1 The Maillard reaction and MRP formation

The Maillard reaction (MR) is a multi-staged non-enzymatic browning reaction between the free carbonyl groups of reducing sugars and protein-derived amino groups. The reaction chain can occur both endogenously via biosynthesis at body temperature, and exogenously during the thermal processing of foods (9). Throughout this review, the term “MRPs” is used when referring to the full spectrum of Maillard reaction products, including early- and intermediate-stage compounds relevant to food chemistry and processing. The term “AGEs” is used specifically when referring to the stable end-stage glycation compounds implicated in biological and disease processes. While all AGEs are MRPs, not all MRPs are AGEs (10).

The MR is described through three broadly defined stages, producing a large variety of compounds at every stage that are collectively described as Maillard reaction products (MRPs). In all stages, environmental factors including pH, temperature and availability of precursors influence these reactions and consequentially the configuration and amounts of MRPs created. While the MR is their major pathway, it is worth noting that additional pathways including lipid peroxidation and the polyol pathway have been shown to form MRPs including AGEs (11). Notably, AA has an individual MR pathway and will be discussed separately to MRPs in Section 2.2.

The early stage begins with reducing sugars binding to the ε-amino group of lysine to form Amadori compounds such as fructoselysine (FL). Furosine, a hydrolysis product of FL, is the compound measured as an indicator of early-stage MR activity (12). The early-stage compounds then undergo several steps including dehydrogenation, oxidation and reconfigurations to form reactive intermediates including 5-hydroxymethyl-2-furfural (HMF) but also several reactive α-dicarbonyl compounds including glyoxal, methylglyoxal and 3-deoxyglucosone (3, 13). In the advanced stage, these intermediates form irreversible and stable compounds that are summarized as advanced glycation end products (AGEs). AGEs exist in both free and protein-bound forms depending on whether the Maillard reaction involves free amino acids or peptides and proteins respectively (14). The most abundantly studied compounds are Nε-carboxymethyllysine (CML) and Nε-carboxyethyllysine (CEL). CML is often used as a proxy marker for the total dietary AGE exposure (15). Lysinoalanine (LAL) is not a product of the MR itself, as it is a heat-induced cross-link between two protein groups. It is relevant in the context of dietary MRP as it co-occurs under similar processing conditions and also contributes to reduced lysine availability (16). Notably, over 20 distinct MRPs have been identified to date and reports in the literature include methylglyoxal hydroimidazolone-1, glyoxal hydroimidazolone-1, pyrraline, pentosidine and argpyrimidine amongst others though quantitative data in pet foods remain limited (3, 15).

### 2.2 Acrylamide formation

Acrylamide (AA) is formed through a distinct type of MR pathway between asparagine and reducing sugars such as fructose or glucose and is hence separately classified as “contaminant” not MRP compound in foods (17, 18). Analog to MRPs, it is commonly formed exogenously during food processing conditions of above approximately 120 °C, where free asparagine and reducing sugars react to form the compound (8, 19). AA was classified as neurotoxin and group 2a probable carcinogen for humans by the International Agency for Research on Cancer (IARC) and its content in food is monitored by government agencies around the world, including the European Union (EU), United States, Australia and New Zealand (20, 21). Currently there are no benchmark levels of AA in pet foods.

### 2.3 Factors influencing MRP and AA levels in pet foods

The accumulation of MRPs and AA in pet foods arises from precursors present in the raw ingredients, any pre-processing applied to those ingredients, and the final manufacturing process including storage (12, 22). Amino acids including lysine and arginine combined with reducing sugars are required for AGE formation, while asparagine in combination with starch-derived sugars can give rise to AA. Starchy grains are a commonly used ingredient in thermally processed pet food formulations, including kibbled foods, where starch-gelatinization occurs under high-heat extrusion (23). Thermal exposure during both pre-processing and final formulation drives the interaction between reducing sugars and amino acids to form MRPs and AA, which then carry over into the finished product. Consequently, the complete processing history of a pet food, not only the parameters of the final formulation step, needs to be considered in product design (12).

#### 2.3.1 Traditional pet food processing

Pet food manufacturing relies on few primary thermal processing methods (24). Extrusion is the most common technique for dry pet foods and involves mixing powdered ingredients with water before subjecting the mixture to high temperature and pressure, typically between 80 and 200 °C, to achieve starch gelatinization and microbiological stability. The extrudate is then cut into pieces, dehydrated using hot air, and lastly coated (12, 25). Pelleting notably often operates at considerably lower temperatures than extrusion. Van Rooijen et al. (26) reported 65–90 °C as typical range for pelleting. In this process, conditioned mash is compressed through a shaped opening at low moisture content, forming dry pellets. Many pre-processed ingredients entering the pelleting process, particularly rendered animal co-products such as meat meal, have already accumulated thermal exposure and are likely to contain MRPs and AA. As these ingredients are common to both pelleting and extrusion, the same carry-over of MRPs and AA into the finished product applies to both processes. This places pelleting amongst “traditional processing techniques” despite its low final processing step temperature (12, 24). Retorting is used for moist and canned formats, where raw ingredients, pre-processed ingredients and powders are blended and heated to initiate structural changes in starch and protein, then sealed in containers and subjected to pressurized heat for sterilization, typically at a temperature of 121 °C or higher, to achieve long-term microbiological stability (12). In the context of pet food processing, extrusion and pelleting are often summarized as dry-processed, whereas retorted products are wet-processed (3).

#### 2.3.2 Mild processing

In contrast to traditional methods, mildly processed pet foods are generally subjected to fewer processing steps involving milder cooking conditions at lower temperatures, typically around 90–100 °C, rather than the multiple high-heat stages characteristic of extrusion and retorting (3, 27). These products often require refrigeration or freezing to maintain microbiological stability, as they do not achieve the ambient shelf-life stability imparted by traditional high-heat processing (27). However, thermal processing can reduce lysine bioavailability (12). Nutritional adequacy is nonetheless determined by formulation, and “complete” diets should meet established nutrient profiles, e.g., the Association of American Feed Control Officials (AAFCO), regardless of processing method (28).

#### 2.3.3 Raw and unprocessed

Raw foods include both homemade and commercial formats and undergo minimal or no thermal processing. They are typically composed of raw muscle meat, organs, and bones, and may be supplemented with vegetables or oils. While commercial raw diets may be subject to regulatory requirements such as zero Salmonella tolerance under EU and UK frameworks (29), microbiological risks have still been documented in commercial products (30). Homemade raw diets in particular have been associated with nutritional deficiencies (29). Microbiological contamination and nutritional imbalance are well-documented risks of raw feeding, and the health benefits commonly claimed for these diets largely lack scientific substantiation, as reviewed by Lyu et al. (31). They serve as a useful reference point in studies comparing dietary AGE exposure due to their low or absent thermal processing.

### 2.4 MRP and AA concentrations in pet foods

Measured concentrations of MRPs and AA vary substantially across pet food formats (Table 1). Across studies, wet and canned pet foods consistently contain the highest AGE concentrations (3, 16, 32). The ranking of mildly processed and extruded dry foods is less consistent and varies by marker. In 26 commercial cat foods, total AGE concentrations in mildly cooked and extruded dry diets did not differ, and CML was lowest in extruded dry diets (33), a pattern also evident for CML in Table 1. These comparisons are drawn from commercial products in which differences in processing intensity occur alongside differences in ingredient composition, moisture content and formulation, so the available data do not isolate thermal processing as the sole determinant of the values reported. Acrylamide was detected primarily in extruded dry food, with reported values ranging from 13 to 358 μg/kg across studies (34, 35), while AA was not detected in mildly processed foods (3; Table 1). HMF concentrations may be higher in dry foods than wet foods, which could be connected to the higher carbohydrate content and extrusion temperatures involved in extruded dry food production (32), though this has not been consistently observed. Kocadagli et al. (3) did not detect HMF in any of 41 samples analyzed, in contrast to the substantially higher values reported by Van Rooijen et al. (26). This discrepancy may reflect differences in analytical methodology, ingredient composition, processing time and temperature, storage conditions, or time elapsed between processing and testing. The concentrations compiled in Table 1 were determined by chromatographic methods, which underpin the standardized approaches for acrylamide analysis (34). One exception is the acrylamide data obtained by adsorptive stripping voltammetry (35). Oba et al. (32) found no significant correlation between MRP concentrations and nutrient levels in their study, leading them to conclude that other factors, such as cooking method, time, temperature, drying conditions, processing type, moisture content, and ingredient sources (e.g., animal or plant), likely play a role in MRP formation. Estimated daily MRP and AA intakes are elevated in companion animals relative to humans. Adult dogs consuming wet diets are estimated to ingest 1.0 mg/kg body weight (bw)^0.75^/day of CML and 50 mg/kg bw^0.75^/day of HMF, with cats on wet diets estimated at 0.8 and 20 mg/kg bw^0.75^/day, respectively (16). For context, the estimated daily intake of total dietary AGEs in humans ranges from 0.55 to 0.66 mg/kg/day for median exposure, rising to 1.4–1.8 mg/kg/day under high-exposure scenarios, with CML contributing 0.057–0.066 mg/kg/day for median exposure and 0.1588–0.2372 mg/kg/day for high exposure (36). Estimated daily AA intake for dogs fed dry food is approximately 590 ng/kg bw/day, estimated to be approximately four times higher than that of adult humans (25) noting that this comparison is expressed on a body weight rather than metabolic body weight basis.

**Table 1:** Concentration of selected MRPs and AA content in pet and human food.

Food type	Species	Processing	CML mg/kg DM	CEL mg/kg DM	HMF mg/kg DM	Acrylamide μg/kg	Acrylamide EU Benchmark [5] μg/kg
Pet food							
Dry dog food (extruded/kibble)	Dog	Extruded	34 ± 10 [1]; 7.75–20.22 [8]	37 ± 14 [1]	5.08 [2]; ND [1]; 270–1920 [8]	15–69 [3]; 40, 76 [1]; 106–358 [4]; 13 [10]	Not defined
Wet dog food (canned/sterilized)	Dog	Canned/retorted	92 ± 36 [1]; 12.27–54.69 [8]	128 ± 39 [1]	3.27 [2]; ND [1]; 340–3020 [8]	3.9–27.0 [3]	Not defined
Mildly cooked dog food	Dog	Mildly cooked (~90 °C)	69 ± 19 [1]	29 ± 7 [1]	ND [1]	ND [1]	Not defined
Freeze-dried dog food	Dog	Freeze-dried	67 ± 6 [1]	73 ± 54 [1]	ND [1]	ND [1]	Not defined
Dry cat food	Cat	Extruded	6.01–20.72 [8]; 31.30 ± 11.57 [14]	45.90 ± 19.17 [14]	2.33 [2]; 240–1,020 [8]	66–269 [4]	Not defined
Wet cat food	Cat	Canned	26.74–60.54 [8]; 59.50 ± 19.61 [14]	88.10 ± 40.97 [14]	0.22 [2]; 70–2,290 [8]	–	Not defined
Mildly cooked cat food	Cat	Mildly cooked (70–90 °C)	51.67 ± 24.70 [14]	37.83 ± 19.50 [14]	–	–	Not defined
Human food							
Beef (stewed/fried)	Human	Stewed/fried	20.3 ± 4.5 [6]^a^	56.3 ± 11.2 [6]^a^	–	~20 [9]	Not defined
Salmon (fried/baked)	Human	Fried/baked	4.1 [6]^a^	5.8 [6]^a^	–	–	Not defined
Rice (boiled)	Human	Boiled	0.7 [6]^a^	0.2 [6]^a^	–	–	Not defined
Chicken breast (fried)	Human	Fried	0.0235 mg/kg [9]	1.16–1.54 [13]	–	–	Not defined
Potato-based food	Human	Fried/baked	0.0252 mg/kg [9]	0.48 [13]	–	~740 [7]; 71–718 [10]	750
Coffee	Human	Roasted	0.0841 mg/kg [9]	–	900–1,200 [11]	~234 [7]; 338 [10]	400–850
Bread/biscuit	Human	Baked	0.1784 mg/kg [9]	1.28–9.85 [13]	14.8–17.1 [11]	~116–133 [7]; 205 [10]	Bread 50–100; biscuit 350
Baby/infant food	Human	Various	40.0–87.3 mg/kg [12]	0.56–2.34 [12]; 5.30 [13]	0.01–343 [11]	~156 [7]; 164–196 [10]	Baby food 40; biscuit for infants 150

Concentrations of Nε-carboxymethyllysine (CML), Nε-carboxyethyllysine (CEL), 5-hydroxymethyl-2-furfural (HMF), and acrylamide (AA) in selected commercial pet foods and human foods, and European Food Safety Authority (EFSA) Benchmark levels of AA for selected foods. Pet food values are reported on a dry matter (DM) basis unless otherwise noted, whereas human food values are reported on a fresh weight (as-eaten) basis. Values are presented as mean ± SD, mean ± SEM, ranges, or single measurements, as indicated by the source. ND, not detected; –, not measured or not reported. [1] Kocadagli et al. (3), *n* = 41 (fresh 15, kibble 13, wet 10, freeze-dried 2); [2] Oba et al. (32), *n* = 53; [3] Sugita et al., (25), *n* = 51 (dry 30, retort 13, canned 8); [4] Veselá and Šucman (35), *n* = 8 (dog 5, cat 3); [5] EFSA (17); [6] Scheijen et al. (67), *n* = 190; [7] Mousavi Khaneghah et al. (22), meta-analysis of 47 articles comprising 230 studies; [8] van Rooijen et al. (26), *n* = 67; [9] Rasool et al. (19), review; [10] Bebius et al. (34), method validation; [11] Martins et al. (68), review; [12] Zhou et al. (69), *n* = 5; [13] Zhang et al., (36), *n* = 334; [14] Tate et al. (33), *n* = 26 (mildly cooked 6, retorted wet 10, extruded dry 10). ^*b*^Values from [6] and [9] for human foods were originally reported on a fresh weight basis (mg/100 g and μg/kg respectively) and have been converted here to mg/kg for direct comparison. They are not adjusted for moisture content, so remain not directly comparable to pet food values reported on a DM basis, which are inherently higher due to water removal.

Table 1 also presents selected human food categories to contextualize pet food exposure levels. Baby food serves as a particularly relevant comparison, as it represents a thermally processed, single-source diet fed over an extended period. Meta-analysis of AA in baby food reported mean concentrations as high as 156 μg/kg, exceeding the EU benchmark level of 40 μg/kg for cereal-based baby foods and 150 μg/kg for infant biscuits, respectively, established under Regulation (EU) 2017/2158 (17, 22, 37). The European Commission has established AA benchmark levels for several human food categories under this regulation as listed in Table 1, yet no equivalent regulatory benchmarks currently exist for AA or AGEs in pet foods (21). It should be noted that pet food values in Table 1 are reported on a dry matter basis, while human food values are reported on a fresh weight basis; the two are therefore not directly comparable, and pet food values appear inherently higher due to water removal.

Given the repetitive dietary pattern of companion animals described above, these elevated intakes are compounded by limited dietary variety. Taken together, the available data indicate that traditionally processed pet foods contain measurable concentrations of MRPs and AA across multiple compound classes. Whether this dietary exposure translates to adverse health outcomes in companion animals has not been established. Current evidence from human and rodent models is reviewed in the following section.

## 3 Health effects in humans and rodents: current evidence

Companion animals share a substantial chronic disease burden with humans. Neoplastic and metabolic causes of death follow similar age trajectories in humans and dogs, and the proportion of neoplastic deaths is nearly identical in both species (25.3% and 27.4%, respectively) (38). Chronic conditions prevalent in human populations, including diabetes mellitus, obesity and arthritis, are each associated with similarly elevated comorbidity in dogs (38). Specifically Type 1 diabetes was proposed to be studied in a canine animal model, whereas Type 2 does not seem to occur in dogs (39). Additionally, companion dogs develop cognitive dysfunction similar to human Alzheimer's disease, proposing them as a translational model for the disease (40). This shared disease landscape suggests that human and rodent evidence on dietary AGEs and AA may be relevant to companion animals, though AGE clearance mechanisms and endogenous AGE accumulation in dogs and cats may differ from those in humans and rodents, and cross-species parallels should be drawn cautiously.

### 3.1 Dietary advanced glycation end-products (AGEs) in human and rodent models

As described above, AGEs are formed both endogenously, as a consequence of normal metabolism, aging, hyperglycaemia, and oxidative stress, and exogenously through dietary intake of heat processed foods (6, 15). In humans, exogenous dietary AGEs are considered significant contributors to the total body AGE pool, adding to the endogenous AGE pool (15). Several comprehensive reviews have examined the available evidence on endogenous and dietary AGEs and AA, and their potential role in chronic disease states in humans and rodent models (5, 6, 10, 15, 22, 41, 42).

In humans, it has been proposed that two primary mechanisms exert the pathological effects of AGEs. First, AGEs bind to the receptors for AGEs on cell membranes, activating a variety of signaling pathways that lead to triggering oxidative stress and chronic inflammation. While there are a multitude of receptors reported in the literature, the most investigated receptor is the receptor for advanced glycation end-products (RAGE), commonly referred to as RAGE. Second, AGEs directly cross-link tissue proteins, altering their structural and functional properties. These mechanisms operate across multiple organ systems, which explains the breadth of AGE-associated pathology (5, 10, 15). Among the most extensively studied disease areas are diabetes mellitus, cardiovascular diseases (CVDs), neurodegenerative diseases and cancers.

#### 3.1.1 Diabetes mellitus

In diabetes mellitus, AGEs contribute to disease pathology through both direct protein cross-linking and indirect activation of the RAGE signaling cascade, which triggers downstream inflammatory, oxidative, and apoptotic pathways that impair insulin signaling and promote pancreatic β-cell dysfunction (43). This creates a self-reinforcing cycle of inflammation, mitochondrial dysfunction, and progressive insulin resistance (5, 43). In animal models, evidence suggests that dietary AGEs may contribute to diabetes development. In non-obese diabetic mice, which typically develop autoimmune type 1 diabetes by 4–5 months of age, feeding an AGE-restricted diet reduced disease incidence to less than 15%, with protective effects persisting across two subsequent generations (44). Comparable reductions in metabolic dysfunction have been observed in type 2 diabetes model mice fed diets with reduced thermal processing and lower AGE content (5). In a human pilot randomized controlled trial, long-term dietary AGE restriction in older adults with type 2 diabetes was associated with improvements in insulin sensitivity and metabolic markers (45).

#### 3.1.2 Cardiovascular diseases (CVDs)

AGEs are also implicated in cardiovascular diseases (CVDs), with emerging evidence linking AGE accumulation to multiple cardiovascular conditions including coronary artery disease, heart failure, hypertension, valvular heart disease and diabetic cardiomyopathy (41). AGEs contribute to cardiovascular pathology through several mechanisms. Cross-linking of collagen and elastin in blood vessel walls leads to arterial stiffness and cardiac fibrosis, while activation of the AGE-RAGE axis triggers oxidative stress and inflammation (5, 10, 41). AGEs also directly impair cardiovascular cell populations by inhibiting autophagy, inducing apoptosis, disrupting endothelial repair, and promoting fibrosis through myofibroblast transformation (41). AGE modification of plasma lipoproteins increases their atherogenic potential, accelerating deposition in vessel walls, foam cell formation, and plaque development (5, 15). In rat models, high-AGE diets promote endothelial dysfunction and atherosclerosis (5), while dietary AGE reduction lowers RAGE expression and decreases inflammatory adhesion molecules associated with CVD (10). In a rodent study, Verboven et al. (46) demonstrated that healthy rats fed a “Western-style” diet for 18 weeks developed hyperglycaemia, elevated AGE levels, and cardiac impairment. In humans, short-term dietary AGE restriction has been associated with reductions in circulating AGE concentrations and markers of inflammation and oxidative stress, both in healthy subjects and in those with cardio-kidney-metabolic conditions (41). However, dietary AGEs still warrant additional study to fully understand their connection to CVDs. It is important to note that canine cardiovascular disease often differs from the human condition. Dogs rarely develop coronary atherosclerosis, and the most prevalent canine cardiac disease is myxomatous mitral valve disease (MMVD), which is recognized as a translational model for the human condition (47).

#### 3.1.3 Neurodegenerative diseases

AGEs are also implicated in neurodegenerative disease, including Alzheimer's and Parkinson's disease. The role of AGEs in neurodegeneration involves multiple interacting mechanisms, including oxidative stress, mitochondrial dysfunction, accumulation of amyloid-β and phosphorylated Tau protein, and neuroinflammation driven by dysregulation of the AGE-RAGE axis (5, 15). In rodent models, AGEs promote phosphorylation of Tau protein, a recognized early marker of Alzheimer's disease, leading to synaptic damage and impaired memory (5). In a clinical study of older adults without dementia, West et al. (48) reported that higher dietary AGE intake was associated with a faster rate of decline in memory. This represents early human and rodent evidence that dietary AGE exposure may contribute to cognitive impairment as a modifiable contributor, though further investigation is required.

#### 3.1.4 Cancer

AGEs have also been implicated in cancer development, though the evidence is less established than for metabolic (diabetes mellitus) and cardiovascular diseases. AGEs have been detected in several human tumor types and AGE-modified proteins are implicated in cancer cell proliferation, migration, and invasion (6, 15). However, a recent systematic review and meta-analysis found no clinically significant reduction in overall cancer risk associated with high dietary AGE intake in humans, though an association with invasive breast cancer was reported (42). The mechanisms linking dietary AGEs to cancer, including protein damage, aberrant cell signaling, and reduced genetic fidelity, are biologically plausible but require further investigation through prospective studies.

#### 3.1.5 Other disease systems

Beyond these major disease areas, dietary AGE accumulation has been associated with a range of other conditions including chronic kidney disease (CKD), liver disease, joint disease, skin aging, reproductive dysfunction, and intestinal disorders including inflammatory bowel disease (5, 6). While evidence for these conditions is less developed than for diabetes mellitus, the shared underlying mechanisms of AGE-RAGE interaction, oxidative stress, chronic inflammation, and protein cross-linking suggest a shared AGE-driven pathological mechanism.

### 3.2 Acrylamide in human and rodent models

Acrylamide (AA) forms during high-temperature processing of carbohydrate-rich foods via the Maillard reaction (see Section 2.2). Once ingested, acrylamide is rapidly absorbed and converted by cytochrome P450 to glycidamide, which forms DNA adducts and is considered responsible for acrylamide's genotoxic effect (20, 21). AA is classified as a probable human carcinogen (IARC Group 2A), and the European Food Safety Authority (17) has concluded that no safe level of AA intake can be defined. The margin of exposure (MOE), calculated as the ratio of the benchmark dose causing adverse effects in animal studies to the estimated dietary exposure, is used to assess risk from genotoxic carcinogens. A lower value indicates a smaller safety margin, and a MOE of 10,000 or less is considered to indicate a public health concern (49). Estimated dietary acrylamide exposure in European adults yielded MOE values of 1,523–2,126 (8), indicating that current exposure levels are of potential concern.

AA has been associated with carcinogenic, neurotoxic, and reproductive effects in both animal models and humans. In rodent carcinogenicity studies, chronic oral AA administration induces tumors of the thyroid, mammary glands, and testicular mesothelium (7). In a population-level risk assessment of dietary AA exposure in Italy, Mihalache and Dall'Asta (4) estimated cancer incidence in humans attributable to AA at approximately 1.6–3.6 cases per 100,000 individuals annually, with breast cancer representing the highest risk in females and kidney cancer in males. AA is also a well-characterized neurotoxin. In laboratory animals, including rats and mice, chronic AA administration at 0.5–50 mg/kg/day induces progressive neurological effects including ataxia, hind-limb splay, and reduced grip strength, with the No-Observed-Adverse-Effect Level for neurotoxicity established at 0.2–0.5 mg/kg/day (7). The mechanisms underlying AA neurotoxicity involve axonal degeneration, oxidative stress linked to mitochondrial dysfunction, and disruption of multiple neural pathways including those governing apoptosis, autophagy, and neuroinflammation (50). However, the specific metabolic processes of AA still require further study (20, 50).

The limited available evidence on dietary exposure of MRPs and AA in companion animals is reviewed in the following section.

## 4 Health effects in companion animals: current evidence

The evidence reviewed in the preceding section suggests that dietary AGEs and AA are implicated in chronic disease development in humans and rodents, and that dietary restriction may reduce disease markers. While there is no direct causal evidence linking dietary AGEs to disease outcomes in companion animals, a growing body of biomarker and observational data supports the plausibility of shared AGE-mediated disease burden.

Several studies confirm that dietary MRPs are absorbed, metabolized, and excreted by companion animals, and that dietary intake directly influences systemic AGE exposure (Table 2). In observational studies of privately owned animals, Palaseweenun et al. (51) measured urinary free CML, CEL, and LAL, a marker for lysine damage, in dogs and cats fed raw vs. traditionally processed dry food diets. Urinary CML, CEL, and LAL concentrations were 2.03, 2.14, and 3.0 times higher, respectively, in dogs fed traditionally processed food compared to raw-fed dogs (*p* < 0.005). Cats showed a similar pattern, with raw-fed animals excreting significantly less urinary AGEs than those fed dry or mixed processed diets (Table 2). The presence of urinary-free CML, CEL and LAL in raw-fed animals may suggest endogenous AGE formation in dogs and cats (51), though as AGE content was not measured in the raw diets themselves, a dietary contribution cannot be excluded. As an observational study of privately owned animals, dietary fat content and protein profile were not standardized, so contributions from lipid peroxidation (see Section 2.1) cannot be separated from those of the Maillard reaction. Van Rooijen et al. (52) confirmed in cats that daily urinary excretion of FL and CML was positively related to dietary MRP intake (FL *p* = 0.03; CML *p* < 0.01), with minimum apparent absorption ranges of 8%−23% for FL, 25%−73% for CML, and 6%−19% for LAL.

**Table 2:** MRP and AA exposure in pet animals.

Study design	Description	Biomarker	Analytical method	Results	Quantitative outcomes	Reference
4-week latin-square crossover	Purpose-bred healthy adult dogs fed four diets differing in degree of thermal processing (ultra-processed wet, ultra-processed dry, air-dried, mildly cooked)	Plasma AGEs (CML, CEL, MG-H1, GH-1, AP); urinary CML, CEL, LAL; serum sRAGE	LC-MS/MS (AGEs), ELISA (sRAGE)	Higher dietary AGE load from processing is associated with greater circulating AGE burden and altered AGE/sRAGE balance	Dietary AGEs: highest in wet food (2.64 mg/100 kcal). Plasma AGEs: highest in wet food (9.99 nM/50 μl), lowest in mildly cooked (2.50 nM/50 μl, *p* < 0.05). Urinary CML: higher in dry food vs. wet and mildly cooked (*p* ≤ 0.028). AGE/sRAGE ratio: differed across diets (*p* = 0.018)	(27)
12-month longitudinal study	Geriatric dogs comparing extruded kibble vs. mildly cooked food	Serum AGEs (CML, pyrraline); AGE-related metabolomic pathways	UHPLC-MS/MS	Long-term feeding of mildly processed food is associated with sustained reductions in circulating AGEs, consistent with reduced glycation stress	Serum CML and pyrraline: lower with mildly processed food vs. kibble. AGE-related metabolomic signatures: reduced vs. kibble-fed controls	(53)
Cross-sectional spot urine sampling	Privately owned dogs fed raw food (low AGE) vs. dry processed food	Urinary free CML, CEL, LAL (creatinine–standardized)	UHPLC-MS	Higher dietary AGE intake is associated with greater systemic exposure and renal excretion of free AGEs	Urinary CML: 2.03 × higher, dry vs. raw (*p* < 0.005). Urinary CEL: 2.14 × higher, dry vs. raw. Urinary LAL: 3.0 × higher, dry vs. raw. CML and CEL correlated with body weight and neuter status	(51)
Cross-sectional spot urine sampling	Privately owned cats fed raw food, dry processed food, or mixed dry + wet processed diets	Urinary free CML, CEL, LAL (creatinine–standardized)	UHPLC-MS	Lower dietary AGE intake is associated with reduced urinary AGE excretion, indicating diet-dependent systemic AGE burden	Urinary CML: 2.81 × higher dry vs. raw, 2.72 × higher mixed vs. raw. Urinary CEL: 4.33 × higher dry vs. raw, 6.33 × higher mixed vs. raw. Urinary LAL: lower in raw vs. mixed (*p* < 0.005). No significant difference between raw and dry	(51)
Two parallel randomized 36-day latin square designs, quantitative 24-h urine collection	Adult female cats fed 6 commercial dry and 6 commercial moist diets	Urinary FL, CML, LAL	UHPLC-MS	Dietary MRPs from commercial feline foods contribute to the endogenous AGE pool and potentially to age-related disease processes	FL and CML excretion: positively related to intake in dry (FL *p* = 0.03, CML *p* < 0.01) and moist food (*p* < 0.01). LAL excretion: no significant intake-excretion relationship. Apparent absorption: FL 8%−23%, CML 25%−73%, LAL 6%−19%. Urinary recovery decreased with increasing intake	(52)
Cross-sectional environmental exposure study	Urine collected from pet dogs and cats in the U.S., assessing volatile organic compounds and acrylamide exposure	Urinary VOC metabolites, including AA metabolite AAMA	HPLC-MS/MS	Pets are substantially exposed to acrylamide and other VOCs, likely from environmental and food sources, with exposure levels suggesting potential health concern	AAMA (dogs): geometric mean 36.6 ng/ml, 77% exceeded *HQ* > 1, all exceeded the screening-level cancer risk benchmark (10^−6^). AAMA (cats): geometric mean 11.1 ng/ml, 50% exceeded *HQ* > 1, all exceeded the screening-level cancer risk benchmark. ∑VOCM: 2,280 μg/g creatinine (dogs), 847 μg/g creatinine (cats)	(59)

Summary of feeding trials and exposure studies investigating advanced glycation end products (AGEs) and acrylamide (AA) in dogs and cats. Studies include feeding trials and one exposure study in pet animals (Cats and Dogs). Measured biomarkers include circulating and urinary AGEs: Nε-carboxymethyllysine (CML), Nε-carboxyethyllysine (CEL), methylglyoxal-derived hydroimidazolone (MG-H1), glyoxal-derived hydroimidazolone (GH-1), argpyrimidine (AP); the protein cross-link lysinoalanine (LAL), early-stage Maillard reaction products [MRPs; furosine (FL)], soluble receptor for advanced glycation end products (sRAGE), and the acrylamide metabolite N-acetyl-S-(2-carbamoylethyl)-L-cysteine (AAMA). Values may include medians, ranges, or statistical comparisons as specified in the original studies. HQ, hazard quotient; VOCM, volatile organic compound metabolites; UHPLC-MS/MS, ultra-high-performance liquid chromatography with tandem mass spectrometry; UHPLC-MS, ultra-high-performance liquid chromatography with mass spectrometry; HPLC-MS/MS, high-performance liquid chromatography with tandem mass spectrometry; LC-MS/MS, liquid chromatography with tandem mass spectrometry; ELISA, enzyme-linked immunosorbent assay.

Controlled dietary studies in dogs provide further evidence. Bridglalsingh et al. (27) fed healthy adult dogs four diets differing in degree of thermal processing in a 4-week Latin-square crossover design. Total plasma AGEs were highest in dogs fed traditionally processed wet food and lowest in those fed mildly processed (fresh) food (median 9.99 vs. 2.50 nM/50 μl; *p* < 0.05). Urinary CML was higher in dogs fed traditionally processed dry food compared to wet and mildly processed diets. Yamka et al. (53) compared geriatric dogs fed traditionally extruded kibble vs. mildly processed food over 12 months and reported that dogs fed mildly processed food had lower serum CML and pyrraline concentrations and reduced AGE-associated metabolomic signatures, consistent with sustained reductions in glycation stress. In both studies the diets differed in composition as well as in degree of thermal processing, so the observed biomarker differences cannot be attributed to processing alone. Dry diets in particular contain a higher proportion of carbohydrate than wet or fresh formats. Because reducing sugars are substrates for the Maillard reaction (see Section 2.1), carbohydrate contributes to MRP formation during manufacture, and postprandial glycaemic and insulin responses also vary with diet composition (54, 55). Composition and processing intensity therefore cannot be separated as contributors to the observed differences. Valle et al. (56) measured serum concentrations of CML, pentosidine, methylglyoxal, advanced oxidation protein products, and malondialdehyde in cats with chronic kidney disease (CKD) and found significantly elevated levels of these glycation and oxidative stress markers in CKD cats compared to healthy controls, identifying them as uraemic toxin biomarkers in feline CKD. These findings parallel human observations of AGE accumulation in diseased renal tissue and raise the question of whether dietary AGE exposure may contribute to similar pathological consequences in companion animals. Hiney et al. (57) compared serum metabolites in dogs fed raw vs. kibble diets and found no significant differences in inflammatory markers. Jimenez (58) found no correlation between plasma AGE concentrations and body mass or age in domestic dogs or wild canids, suggesting that baseline AGE formation may be an evolutionarily conserved trait.

Acrylamide exposure in companion animals has also been measured directly. Sugita et al. (25) estimated the daily acrylamide intake by dogs at 590 ng/kg/day, approximately four times higher than estimated human intake. Pal and Kannan (59) measured urinary AA metabolites in dogs and cats in a cross-sectional environmental exposure study. The geometric mean urinary concentration of acrylamide metabolite N-acetyl-S-2-carbamoylethyl-cysteine (AAMA), the primary AA metabolite, was 36.6 ng/ml in dogs and 11.1 ng/ml in cats. In that study, 77% of dogs and 50% of cats exceeded the hazard quotient threshold of 1 for non-carcinogenic effects, and all dogs and cats exceeded the screening-level cancer risk benchmark applied by the authors. Ikeda et al. (60) demonstrated that acrylamide absorption in beagle dogs is rapid and extensive at an administered dose of 1 mg/kg/day over 3–4 weeks. The conversion rate of AA to its carcinogenic metabolite glycidamide remains unknown for dogs (3). Cytochrome P450 2E1, the enzyme principally responsible for this conversion, has been characterized in dogs (61) and cytochrome P450 2E polymorphism described in cats (62), but its catalytic efficiency toward acrylamide has not been determined in either species. Satchell and McLeod (63) reported that dogs are susceptible to AA-induced neuropathy, with oral administration at 7 mg/kg/day producing first clinical signs after 20–28 days and established neuropathy after 6–8 weeks. Le Roux-Pullen and Lessing (64) reported a case of acute AA neurotoxicity in Labrador puppies that developed ataxia, hypermetria, and convulsions after consuming burnt maize porridge, which the authors suggested may have been consistent with AA toxicity, but AA content was not established in the food sample. While the estimated daily canine intake of 590 ng/kg bw/day (25) is well below the No-Observed-Adverse-Effect Level for neurotoxicity in rodents (0.2–0.5 mg/kg bw/day) (7), this comparison does not account for the cumulative effect of sustained daily exposure to high-heat processed food, nor for potential species differences in acrylamide metabolism and susceptibility.

## 5 Discussion

As outlined in the Introduction, companion animals commonly rely on traditionally processed food as a dietary staple over prolonged periods. Adverse health outcomes attributable to dietary MRPs or AA have not been demonstrated in companion animals, and neither has their absence, as the question has not been directly investigated in these species. Human and rodent evidence indicates that dietary AGEs are associated with diabetes, cardiovascular disease, neurodegeneration, and specific cancers, while AA is associated with carcinogenicity and neurotoxicity, through mechanisms that continue to be investigated. Dietary restriction of AGEs has been shown to reduce disease markers in humans and rodents (44, 45). Dogs share similar age-related disease trajectories (38), and controlled dietary studies indicate that diet type modifies systemic AGE exposure (27, 53). Available data suggest that mildly processed diets may be associated with lower MRP and AA levels compared to traditionally processed formats, though the evidence base remains limited and this warrants further investigation before conclusions can be drawn. Any such comparison must also account for the trade-offs of reduced thermal processing, including a smaller microbiological safety margin, dependence on cold-chain distribution, and the resulting limits on affordability and accessibility. Lower MRP and AA content is therefore one consideration among several in diet selection rather than a basis for recommending a dietary category.

Despite these advances, substantial evidence gaps remain. No long-term controlled feeding trials have measured health outcomes against dietary MRP, AGE or AA intake in dogs or cats. Sample sizes in existing studies are small. No validated safe intake thresholds for MRPs or AA exist for companion animals. EU Regulation 2017/2158 establishes AA benchmarks for human foods, but as noted in Section 2, no equivalent standards exist for pet foods in any jurisdiction. Where exposure cannot be eliminated, the “As Low As Reasonably Achievable” (ALARA) principle, widely applied in food contaminant regulation, could provide a practical framework for setting manufacturing targets in the absence of established safe thresholds. Individual MRP and acrylamide concentrations in pet foods may appear low relative to doses producing acute toxicity in rodent studies (see Section 3.2) but pets may consume the same thermally processed food regularly for years, resulting in sustained daily exposure that exceeds the duration of existing experimental models.

The more relevant comparison may therefore be the long-term cumulative burden rather than the single-dose toxicity threshold or short-term exposure. That burden is not limited to MRPs and AA. Microplastics have recently been reported in commercial dry pet foods in European and South American markets (65, 66), so a single processed staple may carry several unrelated contaminant classes under the same pattern of chronic exposure. Whether their combined effect exceeds that of any one compound has not been examined in dogs or cats, and lies outside the thermal processing mechanism that defines this review. Given the plausible shared pathophysiology and the absence of species-specific safety data, a precautionary approach to pet food safety standards and veterinary nutrition guidance may be warranted.

## 6 Conclusion and future directions

Traditionally processed pet foods expose companion animals to elevated MRPs and AA over prolonged periods, and these compounds are absorbed and measurable in serum and urine. Whether this exposure results in adverse health outcomes has not been established. Priority research needs include prospective cohort studies linking dietary MRP exposure to clinical outcomes in dogs and cats and the development of species-specific biomarkers. Long-term controlled feeding trials would provide the strongest evidence but remain costly and logistically challenging to conduct. Dogs with canine cognitive dysfunction may serve as translational models to investigate whether dietary AGE restriction can mitigate neurodegeneration. Establishing regulatory frameworks for MRPs and AA in pet foods, potentially adopting the “As Low As Reasonably Achievable” (ALARA) principle, could be considered as a policy direction. Mildly processed diets may offer a route to reducing dietary exposure, though this remains to be established and must be weighed against the microbiological, storage and accessibility considerations that accompany reduced thermal processing.
